# Improved bactericidal efficacy and thermostability of *Staphylococcus aureus*-specific bacteriophage SA3821 by repeated sodium pyrophosphate challenges

**DOI:** 10.1038/s41598-021-02446-1

**Published:** 2021-11-25

**Authors:** Hyo Ju Choi, Minsik Kim

**Affiliations:** Laboratory of Molecular Food Microbiology, Department of Food and Nutrition, Brain Korea 21 FOUR, Institute of Symbiotic Life-TECH, College of Human Ecology, Yonsei University, Seoul, 03722 Republic of Korea

**Keywords:** Bacteriophages, Phage biology, Applied microbiology

## Abstract

As antibiotic resistance is being a threat to public health worldwide, bacteriophages are re-highlighted as alternative antimicrobials to fight with pathogens. Various wild-type phages isolated from diverse sources have been tested, but potential mutant phages generated by genome engineering or random mutagenesis are drawing increasing attention. Here, we applied a chelating agent, sodium pyrophosphate, to the staphylococcal temperate *Siphoviridae* phage SA3821 to introduce random mutations. Through 30 sequential sodium pyrophosphate challenges and random selections, the suspected mutant phage SA3821^M^ was isolated. SA3821^M^ maintained an intact virion morphology, but exhibited better bactericidal activity against its host *Staphylococcous aureus* CCARM 3821 for up to 17 h and thermostability than its parent, SA3821. Sodium pyrophosphate-mediated mutations in SA3821^M^ were absent in lysogenic development genes but concentrated (83.9%) in genes related to the phage tail, particularly in the tail tape measure protein, indicating that changes in the tail module might have been responsible for the altered traits. This intentional random mutagenesis through controlled treatments with sodium pyrophosphate could be applied to other phages as a simple but potent method to improve their traits as alternative antimicrobials.

## Introduction

As drug-resistant bacteria have been emerging and spreading around the world, it critically considered as severe concern especially in clinical settings^1^. Drug-resistant variants of infectious pathogens, such as methicillin-resistant *Staphylococcus aureus* (MRSA) and vancomycin-resistant *S. aureus* (VRSA), have emerged and spread in the community as well as hospitals and healthcare facilities, further endangering public health^2,3^. The development of novel chemotherapeutics is costly and time consuming^4^, so the development of new antibiotics is not expected to be a complete countermeasure to antimicrobial-resistant pathogens in the coming post-antibiotics era. Consequently, researchers are working to identify alternative antimicrobials.

Bacteriophages (phages) are viruses that are obligatory parasites of bacteria or archaea^5^. Most known phages that infect bacteria are classified into the order of *Caudovirales*, which consist of an icosahedral or prolate head and a long or short tail^6^. Tailed phages belonging to the family *Siphoviriade* with a long, flexible tail use several tail proteins to recognize specific host bacteria. The binding of phage tail to the host receptors on bacterial cell surfaces triggers the phage to undergo structural changes, that allow phage to translocate its genome from its head into the host cytoplasm^7^. During this process, a tail-associated lysin (Tal) punctures the bacterial cell wall^8^ after which phage tail tape measure proteins (TMP) form a channel-like structure into the cytoplasm to allow for genome entry^9^. After genome replication and expression within the infected host, newly synthesized progeny phages escape from the host by tearing through the bacterial cell walls.

Phages have gained a great interest as one of the promising alternative antimicrobial agents with several advantages over conventional antibiotics^10^. The abundancy at nature (approximately 10^32^ phages on the planet) and their intrinsic traits infecting and lysing host bacteria make phages reliable candidates to fight with the pathogens^11^. Unlike to antibiotics, phages also specifically affect on host bacteria and hardly disrupt a normal microflora^12^. Phages propagate themselves as long as the viable host bacteria exist, allowing for self-dosing during therapy and remediation^13^. Generally, the use of lytic virulent phages is mandatory for these purposes, because temperate phages can stay in the infected host bacterium as prophages without causing immediate host lysis. Indeed, several previous reports demonstrated the used of lytic phages as novel alternative antimicrobials in various fields, including human^13^ and veterinary medicine^14^, agriculture^15^, and the food industry^16,17^.

However, there are some limitations to phage application. Phage activity can be hindered by characteristics of their external environment, such as temperature and acidity^18^. Phages often have insufficient bactericidal efficacy, and sometimes can only be used to control a narrow range of bacteria^19^. Attempts have been made to engineer phages that are not limited by these shortcomings, such as developing recombinant phages to enhance their adsorption ability^20^ and improving their pH resistance^21^. However, these phage genome engineering methods require knowledge of how to manipulate genes, knowledge about phage genomes and associated vectors, and related skills^22^. Furthermore, the use of genetically engineered organisms developed by artificial and intentional genetic recombination is still controversial among the public^23^.

One way to simply and rapidly obtain phage variants without genetic engineering is by treating them with chemicals, such as chelating and alkylating agents^24,25^. Sodium pyrophosphate, a polyanionic chelating agent, destabilizes phage virions by reacting with cations on the surface of phage particle. It tightly binds to the subunits of phage head protein, distorting the head structure, and consequently causing DNA to leak from the head^26^. If phages have shorter genomes caused by the spontaneous loss and mutation of some DNA regions, they may experience less internal head pressure caused by the distortion. Thus, these phages hard to destabilized even in the presence of chelating agents, and can maintain their infectivity. Indeed, after repeated treatments with chelating agents, some phage subpopulations lost dispensable DNA regions from their genomes, such as *Streptomyces* phage ΦA7^27^, *Staphylococcus* phages Φ88 and Φ35^28^, and *Lactobacillus* phage A2^29^.

In this study, the staphylococcal temperate *Siphoviridae* phage isolates SA3821 and SA3956 were subjected to the 30 rounds of sodium pyrophosphate challenges with no selection criteria to produce random mutant variants. The antimicrobial traits and genome of obtained mutant phage SA3821^M^ were analyzed compared with parent phage SA3821, and the involvement of tail mutations in improved bactericidal efficacy and increased thermostability of the SA3821^M^ was suggested and discussed.

## Results

### Sodium pyrophosphate challenges against two staphylococcal phages

**Figure 1:**
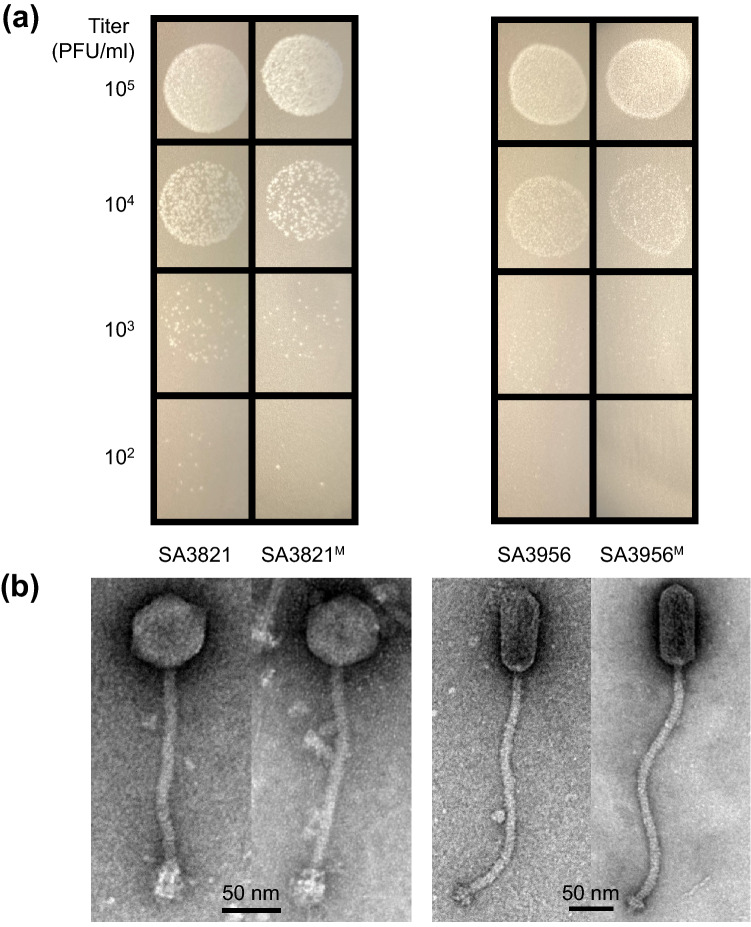
Morphologies of parent and mutant phages. (**a**) Diluted phage solutions (SA3821, SA3821^M^, SA3956, and SA3956^M^) with indicated titers spotted on the lawns of host *S. aureus*. After overnight incubation, formed plaques were compared. The figures are representative of three independent experiments. (**b**) Concentrated phages on carbon-coated copper grids negatively stained with 2% aqueous uranyl acetate. Virion morphology was observed via TEM. Scale bars, 50 nm. Representative images obtained from three biological replicates are shown.

The bacteriophages SA3821 and SA3956 were isolated from environmental samples using the *S. aureus* strains CCARM 3821 and CCARM 3956 as host bacteria, respectively. The plaque morphology and host range were completely different from each other, suggesting that these two phages were distinct (Fig. 1a and Supplementary Table S1). Anticipating the alteration of phage phenotypes, each of the purified phages was subjected to 30 sequential sodium pyrophosphate challenges to generate the random mutant phages as described in “Materials and methods” section and Supplementary Fig. S1. During challenges, the surviving SA3821 and SA3956 phages from single plaques on the host bacterial lawn were randomly isolated and subjected to the next challenge. None of the selection criteria in plaque picking was considered because we intended to be unbiased toward the obtaining of certain mutation(s) but to get any mutations accumulated during the repeated challenges. After 30 challenges, the surviving phages with 100% survival rate under the 400 mM of sodium pyrophosphate treatment were morphologically not distinguishable from each other. We thus isolated a suspected mutant phage from a random single plaque and designated it as SA3821^M^ and SA3956^M^, respectively. The plaque morphology of the parent and mutant phages were not significantly different. SA3821^M^ and its parent SA3821 both formed clear plaques and SA3956^M^ and its parent SA3956 both formed tiny turbid plaques (Fig. 1a).

Transmission electron microscope (TEM) analysis also showed no significant morphological differences between the parents and the mutants. Both SA3821 and SA3956 had the typical morphological characteristics of the family *Siphoviridae*, namely with a virion head and a long, flexible tail (Fig. 1b)^30^. However, they had different head structures. SA3821 had an icosahedral head with a diameter of 54.80 ± 0.87 nm while SA3956 had a prolate head with a length of 93.78 ± 2.90 nm. The morphological characteristics of the mutant phages were similar to their parents. SA3821^M^ had a head diameter of 54.81 ± 1.39 nm and SA3956^M^ had a head length of 94.08 ± 0.64 nm. The phage tail lengths also did not significantly differ between the parents and mutants. SA3821 tail length was 188.70 ± 6.79 nm while SA3821^M^ tail length was 187.93 ± 1.80 nm. SA3956 tail length was 272.39 ± 7.32 while SA3956^M^ tail length was 279.47 ± 3.82 nm.

### Enhanced bactericidal activity of the mutant phage SA3821^M^

**Figure 2:**
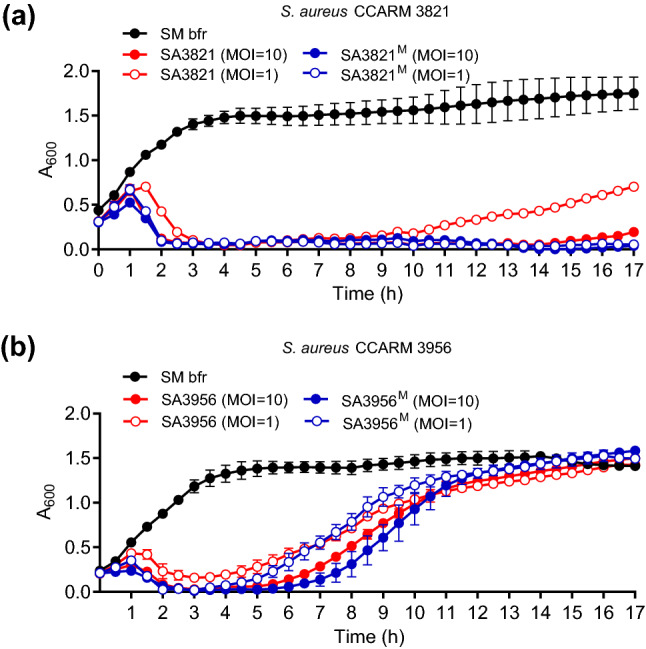
Bactericidal activities of parent and mutant phages. Growth of host *S. aureus* (**a**) CCARM 3821 and (**b**) CCARM 3956 was periodically monitored for 17 h after treatment with (**a**) parent SA3821 and mutant SA3821^M^ and (**b**) parent SA3956 and mutant SA3956^M^ at the indicated MOIs. SM buffer (SM bfr) instead of phages was added as a negative control. Experiments were conducted independently in triplicated and the mean and s.d. are represented.

To determine whether the parent and mutant phages had different efficacies at inhibiting bacterial growth, in vitro bacterial challenge assays were conducted. Both parent phages SA3821 and SA3956 rapidly and significantly inhibited host growth. The growth rates of *S. aureus* CCRAM 3821 and 3956 began to sharply decrease within 1 h (Multiplicity of infection [MOI] = 10) and 1.5 h (MOI = 1), respectively, in the presence of SA3821 and SA3956, respectively (Fig. 2). This growth inhibition of *S. aureus* CCRAM 3821 by SA3821 was maintained for up to 8 h (MOI = 1) and 14 h (MOI = 10), but soon after, phage-insensitive bacterial populations began growing. Similarly, growth resumed in the SA3956-infected *S. aureus* CCRAM 3956 culture after 3.5 h (MOI = 1) and 4.5 h (MOI = 10), which was sooner than for SA3821.

However, the *S. aureus* CCRAM 3821 treated with SA3821^M^ exhibited a different growth pattern than when it was treated with the parent phage SA3821. SA3821^M^ exhibited persistently strong inhibitory activity against the host growth without causing a phage-insensitive population to emerge (Fig. 2a). This enhanced antibacterial activity was pronounced even at low MOI. Although the host growth had gradually recovered 8 h after treatment with the parent phage SA3821 (MOI = 1), no host re-growth occurred after treatment with SA3821^M^ at the same concentration for up to 17 h. This result was reproduced with propagated progenies of SA3821^M^, suggesting that repeated challenges with sodium pyrophosphate genetically and phenotypically changed SA3821 into SA3821^M^. In contrast, host growth inhibition pattern and host growth resumption timing of SA3956^M^ treatment were not significantly different from those of SA3956 (Fig. 2b). Thus, SA3821 and SA3821^M^ were further comparably analyzed through subsequent characterization while SA3956 and SA3956^M^ were not.

### Both SA3821 and SA3821^M^ recognize staphylococcal wall teichoic acid as a host receptor

**Figure 3:**
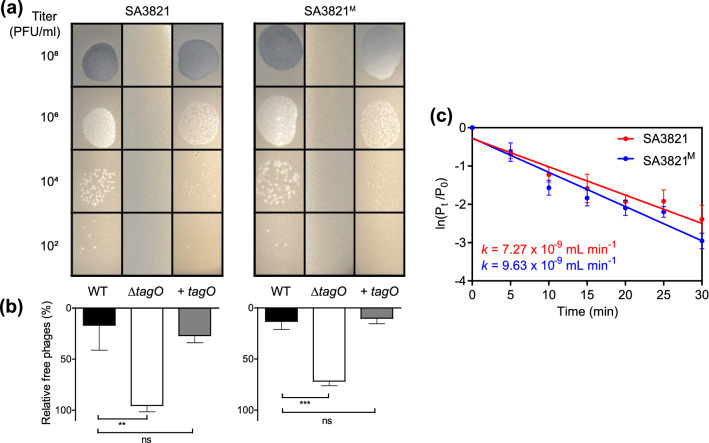
Both SA3821 and SA3821^M^ adsorbed to the host *S. aureus* cells via recognition of WTAs. (**a**) Phage spotting assay results and (**b**) phage adsorption assay results with *S. aureus* RN4220 wild-type (WT), *∆tagO*::*erm* mutant (∆*tagO*), and *∆tagO*::*erm*-complemented strain (+ *tagO*). The numbers of unbound free phages against each host strain were expressed in percentages compared to the numbers of initial inoculated phages. **(c)** Adsorption kinetics of parent SA3821 and mutant SA3821^M^. Host *S. aureus* CCARM 3821 was infected with phages, and unadsorbed virions in the culture supernatant were enumerated periodically through plaqueing assays. Results are expressed as mean and standard error of the mean. **P < 0.01; ***P < 0.001; *ns* not significant.

Because SA3821 and SA3821^M^ interacted differently with host *S. aureus* CCARM 3821 cells during the bacterial challenge assays, we first determined whether the host ranges were different. We found that both the parent and the mutant phages infected the same ranges of *S. aureus* strains, which included 15 of the 32 strains tested, 2 of which were MRSA strains (Supplementary Table S1). Thus, we next examined whether the two phages had recognized the host for different reasons at the early stage of infection. To determine the host receptor for phage SA3821, the susceptibility of *S. aureus* RN4220 mutants lacking various components on their surfaces was determined through phage spotting assay. All mutants tested except the *tagO* deletion mutant supported the formation of SA3821 plaques (Supplementary Table S1) and a *tagO* complemented strain caused phage susceptibility to return to its original levels (Fig. 3a). The same results were obtained with the mutant phage SA3821^M^, indicating that both SA3821 and SA3821^M^ infection were dependent on the presence of *tagO* in the host *S. aureus*.

The gene product of *tagO* is involved in the biosynthesis of wall teichoic acid (WTA) in *S. aureus*^31^. We further determined staphylococcal WTA was the host receptor of SA3821 through an adsorption assay. As shown in Fig. 3b, the proportion of unbound free SA3821 virions was significantly increased with the *tagO* mutant compared to the *S. aureus* RN4220 wild-type (WT), but that was restored to the WT level with the *tagO* complemented strain. The mutant phage SA3821^M^ exhibited the same pattern (Fig. 3b), indicating that the parent SA3821 and mutant SA3821^M^ both recognize the staphylococcal WTA as the host receptor for their initial adsorption to host cells.

Although the two phages utilized the same host receptor, the differences in their adsorption rates may have caused the results of their in vitro challenge assays to differ. Five minutes post-infection, 49% of the SA3821 phages had been adsorbed while 51% of the SA3821^M^ had been, which are similar rates. However, more SA3821^M^ phages were adsorbed over time than SA3821 phages (Fig. 3c), and the mutant SA3821^M^ exhibited a 132% higher adsorption constant (*k*) than its parent SA3821.

### Accelerated progeny synthesis but reduced burst size in SA3821^M^

**Figure 4:**
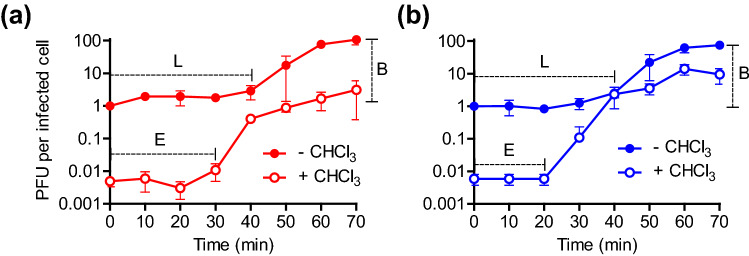
One-step growth curves for (**a**) parent SA3821 and (**b**) mutant SA3821^M^. The replicative characteristics (i.e., E, eclipse period in min; L, latent period in min; and B, burst size in PFU/infected cell) of each phage were determined during one round of phage propagation in single *S. aureus* CCARM 3821 cells. Biological three independent experiments were conducted and the mean and s.d. are shown. CHCl_3_; chloroform.

After phage adsorption, phage genome is injected into the host cell and infectious viral progenies are newly synthesized within the host cell. To determine whether the mutant SA3821^M^ and its parent SA3821 behaved differently at this stage, their one-step growth curves were compared. The two phages were well propagated within the host cell, but they had different population growth pattern. The eclipse period, which is the amount of time that passed after infection that infectious progeny first clearly appear within an infected host cell, was approximately 30 min for of SA3821, but that of mutant SA3821^M^ was approximately 20 min (Fig. 4a, b). The latent period, which is the amount of time that passed after infection that newly synthesized virions begin to be released from the host to the surrounding environment, was approximately 40 min for both phages. Interestingly, however, the mutant phage SA3821^M^ released less amount of progenies after the infection of a single cell than the parent phage SA3821. The burst size of the parent SA3821 was 107 PFU per infected cell but that was approximately 30% lower at 75 PFU per infected cell for mutant SA3821^M^ (Fig. 4).

### Increased thermostability in the mutant phage SA3821^M^

**Figure 5:**
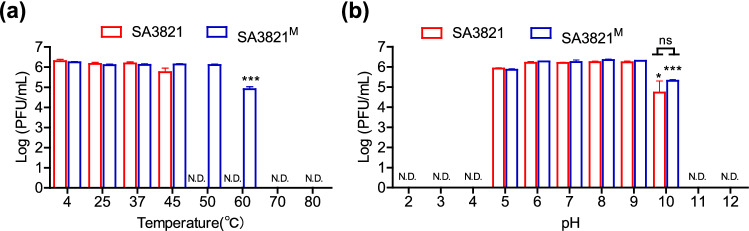
The stability of phages SA3821 and SA3821^M^ under various (**a**) thermal and (**b**) pH conditions. Each phage was exposed at the indicated temperature at a pH of 7.0 or pH at 25 °C for 1 h. Statistical differences were determined by comparing values (**a**) at 4 °C and (**b**) at pH 7. Results of three independent biological triplicates are shown as the mean and s.d. *N.D.* not detected, *ns* not significant; *P < 0.05; ***P < 0.001.

To compare the tolerance of parent and mutant phages under stressful external conditions, virions were exposed to various heat and pH conditions. The parent phage SA3821 lost its infectivity when exposed to temperatures of at least 50 °C for 1 h, indicating that it was vulnerable to thermal stress (Fig. 5a). However, the mutant SA3821^M^ was stable and exhibited infectivity even at 60 °C, though the population did decrease by 1.2-log PFU/mL. At temperatures of at least 70 °C, both SA3821 and SA3821^M^ were inactivated within 1 h.

In the stability test of various pH conditions, both parent and mutant phages showed similar results. They could infect their host *S. aureus* CCARM 3821 cells after exposure to pH 5–10 for 1 h, though there were significantly fewer infectious phages after exposure to pH 10 than pH 7 for both phages. There was 5.31 ± 0.05 log PFU/mL of SA3821^M^, which was greater than the 4.72 ± 0.58 log PFU/mL of its parent SA3821, but this difference was not statistically significant (Fig. 5b). Highly acidic conditions of pH 2–4 and basic conditions of pH 11–12 were equally detrimental to the mutant and the parent.

### Random mutations concentrated in a gene encoding tail tape measure protein in SA3821^M^

To determine the genetic reasons for the characteristics of SA3821^M^ that differed from its parent, such as its increased bactericidal activity and thermotolerance, the complete genome sequences of SA3821 and SA3821^M^ were analyzed. The genome of phage SA3821 (Genbank accession number: MW846279) consisted of dsDNA of 43,417-bp with 34.24 GC%, 65 ORFs, and no tRNA genes. Of the 65 predicted ORFs, 36 (55%) encoded hypothetical proteins with unknown functions. The other ORFs were grouped according to whether the putative function of the encoded gene products was related to structure, packaging, DNA regulation, DNA manipulation, or cell lysis (Supplementary Fig. S2 and Table S2). The group of structural proteins included phage major and minor capsids, baseplate, tail, and tail TMPs. A protein containing glucosaminidase domain was predicted to be adjacent to the baseplate gene, which might function as a Tal to facilitate the digestion of host cell walls during phage DNA translocation to the host^32^. The ORFs holin and endolysin, which are associated with host bacterial cell lysis at the stage of progeny release, were grouped in the host lysis category. Proteins related to regulating gene expression, such as the transcriptional activators RinA and RinB, putative *cro*/*cI*-like repressors, and anti-repressors were grouped in the DNA regulation category. Endodeoxyribonuclease, recombinase, single-stranded DNA-binding proteins, and DnaD domain-containing proteins were grouped in the DNA manipulation category.

Mutant phage SA3821^M^ possessed a genome of 43,396-bp with 34.25 GC%, which was 21-bp shorter than that of its parent SA3821, but the same numbers and arrays of ORFs were predicted for it as for its parent SA3821. A total of 306 nucleotide substitutions from its parent SA3821 that led to 92 amino acid (a.a.) changes were found in the SA3821^M^ genome. None of these substitutions were present in any intergenic regions but rather were focused in the coding sequences of 9 different genes: endodeoxyribonuclease, the DnaD domain-containing protein, TMP, the tail assembly chaperone, the major tail protein, the putative tail component, and three hypothetical proteins (Table 1 and Supplementary Fig. S3). Deletions of 21 base pairs were also found within the TMP gene in SA3821^M^. However, none of these nine SA3821^M^ proteins was expected to be truncated because all of the nucleotide substitutions resulted in silent or missense mutations and no nonsense mutations. The 21-bp deletions also did not cause TMP translation to stop prematurely.

**Table 1:** The number of mutations in the mutant phage SA3821^M^.

ORFs (predicted gene product)	Mutations at the nucleotide level		Mutations at the amino acid level		
	Substitutions	Deletions	Missense mutations	Silent mutations	Deletions
Total	306 (100.0%)	21	92 (100.0%)	166	7
ORF 15 (hypothetical protein)	8 (2.6%)	–	4 (4.3%)	1	–
ORF 16 (endodeoxyribonuclease)	29 (9.5%)	–	10 (10.9%)	9	–
ORF 18 (DnaD domain-containing protein)	12 (3.9%)	–	5 (5.4%)	5	–
ORF 51 (tail tape measure protein)	206 (67.3%)	21	62 (67.4%)	112	7
ORF 52 (hypothetical protein)	7 (2.3%)	–	–	7	–
ORF 53 (tail assembly chaperone)	3 (1.0%)	–	1 (1.1%)	2	–
ORF 54 (major tail protein)	20 (6.5%)	–	6 (6.5%)	13	–
ORF 55 (hypothetical protein)	13 (4.2%)	–	3 (3.3%)	10	–
ORF 56 (putative tail component)	8 (2.6%)	–	1 (1.1%)	7	–

**Figure 6:**
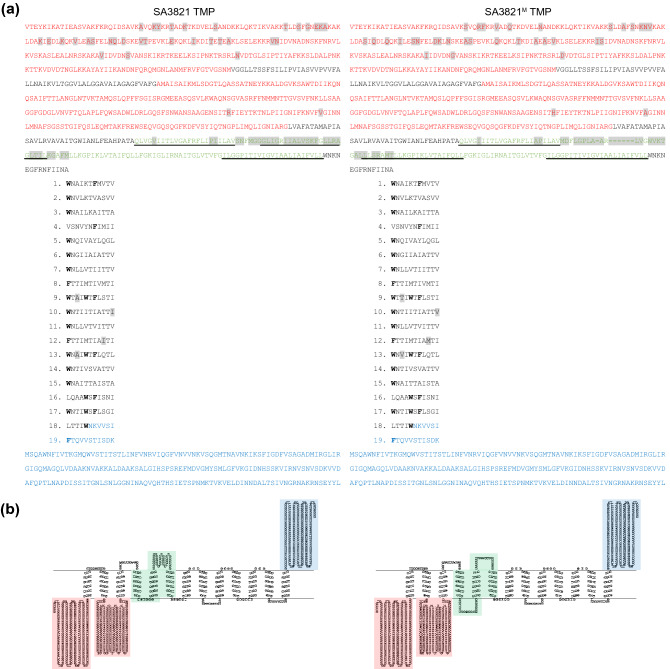
Amino acid sequence and topology predictions for SA3821 TMP and SA3821^M^ TMP. (**a**) TMP amino acid sequences for SA3821 and SA3821^M^ with 19 tandem repeats indicated numerically. a.a. deletions are indicated by hyphens. The positions of a.a. substitutions are indicated with a gray background. The predicted N-terminal, C-terminal hydrophilic, and changed central hydrophobic regions are indicated by red, blue, and green, respectively. Sequences of fourth-to-sixth transmembrane-spanning regions are underlined. (**b**) The topology model of TMP with the color-highlighted as in panel (**a**).

More than 80% of the nucleotide substitutions and deletions located in a module that consist of 6 consecutive genes related to the phage tail (i.e., ORF 51–56). The others were found in another module containing ORF 16 for endodeoxyribonuclease and ORF 18 for DnaD domain-containing proteins. However, they were not predicted to be involved in the altered traits of SA3821^M^ because they located outside of important functional motifs of each protein (Supplementary Figs. S4 and S5). In particular, approximately 67% of a.a. changes and 7-a.a.s deletion occurred in the SA3821^M^ TMP (Supplementary Table S3). Thus, whether the structural characteristics and topology of TMP in SA3821^M^ were different from those of SA3821 was analyzed. In the level of secondary structure, TMPs of two phages were predicted to be not significantly different because alpha helices were present throughout the proteins except for the C-terminal extremity where a short region of coils and beta-sheet existed (Supplementary Fig. S6). This secondary structure also corresponded with the TMP in phage lambda^33^ and lactococcal phage TP901-1^7^. Furthermore, similar to the other phage TMPs^34^, tandem repeat sequences (19 repeats spaced 11 a.a. apart) containing the aromatic residue markers (i.e., phenylalanine and tryptophan) were identified in the TMPs of both SA3821 and SA3821^M^ (Fig. 6a). All the mutations and deletions except for 4 substitutions in the SA3821^M^ TMP occurred outside the tandem repeats. The 4 substitutions in the repeats also occurred away from the marker residues.

The mutations did not completely reorganize the regional topology of the phage TMP. Both phages were predicted to possess N-terminal and C-terminal hydrophilic regions and a central hydrophobic region, which may represent a membrane-associated domain, in their TMPs (Fig. 6b). The N-terminal hydrophilic region was further divided into two parts by a hydrophobic transmembrane region spanning from residue V263 to G318 of both phages’ TMPs. More than half of missense mutations (35/62) in the SA3821^M^ TMP occurred in the N-terminal hydrophilic regions, of which most occurred at the first one (33/35) (Fig. 6a). The other 27 alterations and deletions occurred in the central hydrophobic region, resulting in a slight topological change in the mutant SA3821^M^ TMP. Accompanying the 7 a.a.s deletion that occurred at the fifth putative membrane-spanning region, a small hydrophilic stretch of 40 a.a.s between the fifth and sixth hydrophobic transmembrane regions in the SA3821 TMP was significantly shorter in the SA3821^M^ TMP while a new short hydrophilic stretch was generated between the fourth and fifth hydrophilic transmembrane regions in the SA3821^M^ TMP (Fig. 6a, b).

The phages SA3956 and SA3956^M^ which showed similar host inhibition activity (Fig. 1b) had the same 44,701-bp dsDNA genomes, indicating that the sodium pyrophosphate challenges did not modify or alter SA3956 genome under the experimental conditions tested.

## Discussion

Sodium pyrophosphate treatments have been applied in some studies to mutagenize temperate phages^28,29,35^. In these studies, alterations in phage plaque clarity and/or inability of host lysogenization were chased as selection criteria during the repeated challenges to obtain the virulent counterparts of the temperate phages. The mutations were largely found in genes related with the lytic-lysogenic decision. For example, a *S. aureus*-specific temperate phage SA13 obtained mutations in the integrase and CI repressor genes after 25 rounds of sodium pyrophosphate treatments, converting to the virulent SA13m that formed clear plaques^35^. Compared to these previous studies, we intended to investigate any random mutations that accumulated during the repeated sodium pyrophosphate challenges to figure out the genetic alterations involved in the phage’s phenotypic trait changes. Thus, we did not set any selection criteria but randomly selected the mutant plaques. This unbiased approach, indeed, resulted in the novel finding of the mutant phage SA3821^M^ which showed improved traits in bactericidal efficacy and thermostability, rather than the previously reported conversion to the virulent phage.

The presence of putative phage repressor and anti-repressor genes suggested the phage SA3821 as a temperate phage (Supplementary Table S2). Indeed, SA3821-specific PCR amplifications revealed the formation of SA3821 lysogens in prophage-cured *S. aureus* RN4220 (Supplementary Fig. S7)^36^, suggesting that the host regrowth in Fig. 2a might be explained with the SA3821 lysogenization.

It is noticeable that more than 67% of mutations of the mutant phage SA3821^M^ were concentrated in the TMP. The number of periodic tandem repeats in the TMP was reported to be linearly proportional to phage tail length^7^. In accordance with that the TMP mutations in the SA3821^M^ did not alter the 11 a.a. periodicity (Fig. 6), no significant changes in the tail length were observed in the mutant SA3821^M^ (Fig. 1b). In a meantime, the TMP N-terminus has been reported to be associated with the assembly between the tail and the head–tail connecter protein and with the inhibition of tail polymerization termination if the tail length is too short^37^. Although more than half mutations in SA3821^M^ TMP were present at the N-terminus (35/62) (Fig. 6a), the phage was apparently intact and normally infectious, indicating that the observed a.a. substitutions were not critical for the above mentioned functions. Similarly, the overall topology and predicted helical structure of the TMP were also unchanged (Fig. 6b and Supplementary Fig. S6), allowing the mutated TMP to exert its normal function in transferring phage DNA to the host cytoplasm. These results suggest that the random mutations that affecting phage infectivity and structural integrity were excluded during the repeated sodium pyrophosphate challenges and subsequent plaqueing, while the tolerable and affirmative mutations improving phage traits were accumulated throughout the challenges.

On the contrary, these mutations might have contributed to and sped up the assembly of progeny phages within infected host cells, because the eclipse period was 10 min shorter in the mutant than its parent while the latent period remained similar (Fig. 4). This result further suggests that the mutations in SA3821^M^ did not alter the expression timing of endolysin and holin but rather affected the assembly efficiency of infectious progeny phages. The missense mutation in *orf53* encoding the tail assembly chaperone might also be related with the accelerated virion assembly.

Various phenotypic changes related to phage tail proteins have been reported in other studies. Phage ΦEP24C infecting *Enterococcus faecalis* exhibited increased adsorption due to a point mutation in a long, flexible, fine tail fiber^38^. Mutations in two putative tail proteins of phage FCV-1.01 were assumed to caused alterations in host range and adsorption efficiency^39^. Similarly, the increased adsorption rate of SA3821^M^ might be a result of enhanced interactions between the SA3821^M^ tail and the host receptor WTA through mutations not only in the TMP but in other tail-associated proteins in the tail module, such as ORF54 for major tail protein and ORF 56 for putative tail component.

In a previous study on the lactococcal phages of the *Sk1* virus group, eliminating 40 a.a. from the TMP C-terminus was demonstrated to partly be the genetic determinant of the phages’ increased heat stability^40^. Although the deleted region was not the same in SA3821^M^ TMP (Fig. 6b and Supplementary Fig. S8), a.a. alterations including the 7 a.a.s deletion in the TMP may have been responsible for the increased thermostability of SA3821^M^. Mutations in tail tubular proteins of the heat-adaptive phages CX5-1 and P-PSG-11–1^41^ and mutations in head-to-tail joining and internal virion proteins of the CAVE-evolved T3 phage variant^24^ were also suggested to be the reason for the increased thermostability. Similar to these examples, not only the TMP mutations but other mutations in the tail-associated genes in the tail module may also have contributed to the increased heat resistance of SA3821^M^.

Although the sodium pyrophosphate treatment is a simple and easy way to obtain the mutant phages, several limitations of this approach need to be carefully considered. Most phage genes are not fully characterized yet and even about half of genes in the mostly studied model phages are still functionally unknown^42,43^. In this context, mutation(s) occurred in the unknown gene(s) could be counterproductive since all the aspects of phages are not assessable by phenotypic experiments. In case of temperate phages, a mutation might unexpectedly affect host gene expression and lead to change in host virulence after the lysogenization. Phage SA3821^M^ also has mutations in 2 unknown hypothetical proteins, implying more complicated influences that we could not characterize in the present study may exist. Another limitation is that the mutations occur randomly throughout the whole phage genome, including genes involved in the phage lytic development. Although infectious phages that lysed the host bacteria and formed noticeable plaques are picked up at each challenge step, detrimental mutations that may cause adverse effects on the lytic development cannot be completely excluded. Therefore, careful validation on the traits of finally obtained mutant phages is critical before the uses.

A careful consideration is also required in the treatment condition. Because sodium pyrophosphate destabilize phage genome by deforming phage head proteins^25^, the different head morphologies of SA3821 and SA3956 (Fig. 1b) is suspected to the one of reasons for differences in their ability to produce mutant phages. Staphylococcal phage ΦA72, which has similar morphology and genome size as SA3956, had been treated with 100 mM sodium pyrophosphate (pH 7.4) for 30 min at 42 °C to generate the lytic variant Φ35^28^. Thus, the experimental conditions used in the present study (i.e., 50–400 mM sodium pyrophosphate [pH 7.5] for 1 h at 37 °C) might be inadequate or insufficient to induce mutations in SA3956. Since the destabilizing effects of the chelating agents are proportional to temperature^26^, it will be worth testing to increase temperatures during sodium pyrophosphate challenges to produce phage SA3956 variants in the further study.

In conclusion, 30 sequential sodium pyrophosphate challenges without any selection criteria can produce the mutant phage SA3821^M^ that had greater infectivity and thermal tolerance than its parent phage SA3821. The mutations were highly concentrated in the tail module (83.9%) and specifically in the TMP (67.3%). These mutations were likely the genetic determinants of the improved properties in SA3821^M^. Being with the adjustment of treatment conditions, sequential sodium pyrophosphate challenges could be used as a simple but potent way to produce active bacteriophage variants possessing improved traits without any sophisticated gene manipulation techniques.

## Materials and methods

### Bacterial strains and growth conditions

The bacterial strains used in this study are listed in Supplementary Table S1. All strains were incubated at 37 °C with aeration in a tryptic soya broth (TSB) medium (Oxoid, Basingstoke, UK). The plaqueing and spotting assays were conducted by overlaying a bottom tryptic soya agar (TSA) plate (1.5% agar) with a top TSA soft agar (TSB with 0.4% soft agar) which is inoculated with the appropriate indicator host strain.

### Bacteriophage isolation, propagation, and stock preparation

Phages SA3821 and SA3956 were isolated from the environment sample (Incheon, South Korea). Samples were mixed with an equal volume of 2X TSB and enriched at 37 °C with aeration for 8 h. The cultured bacterial cells were removed by centrifugation at 9000 g, 4 °C for 10 min and filtration with 0.45 μm-pore filters. Then the filtered supernatant was serially diluted in SM buffer (50 mM Tris-HCl of pH 7.5, 100 mM NaCl, and 10 mM MgSO_4_). The dilutes were spotted on the overlaid TSA plates which is inoculated with the host *S. aureus* CCARM 3821 or CCARM 3956. The single plaques were aseptically picked up and eluted in SM buffer. The elutes were filtered with a 0.22 μm-pore filter and were spotted again on the host bacterial overlaid lawn. This single plaqueing and picking up step was repeated three times to obtain a pure single phage.

Phage propagation was performed as described elsewhere^44^ with some modifications. The host bacterial culture in TSB with 10 mM CaCl_2_ was infected with each phage at the early exponential phase and then incubated at 37 °C. After bacterial lysis, the culture was centrifuged and filtered as described above to remove bacterial debris. The supernatant containing phages was mixed with an NaCl solutions (200 mM, final concentration) supplemented with polyethylene glycol 6000 (10% [v/v], final concentration). Virions were precipitated overnight at 4 °C with gentle shaking. The precipitated phages obtained after centrifugation at 10,000 g, 4 °C for 15 min were resuspended in SM buffer then concentrated via CsCl density gradient ultracentrifugation at 78,500 g, 4 °C for 2 h. The band with concentrated phages was extracted and dialyzed with a dialysis buffer (10 mM NaCl, 10 mM MgSO_4_, 50 mM Tris–HCl of pH 7.5). The prepared phage stocks were stored at 4 °C for further use.

### Sodium pyrophosphate challenges

Phages were repeatedly challenged with sodium pyrophosphate to induce random mutogenesis as previously described^29^ with some modifications (Supplementary Fig. S1). Phages (10^7^ PFU/mL) were treated with 50 mM of sodium pyrophosphate (final concentration; pH 7.5) and incubated at 37 °C for 1 h with gentle shaking. The same volume of Tris-HCl (200 mM, final concentration; pH 7.5) was used as a control treatment. The phages challenged with the sodium pyrophosphate plaqued on the lawns of the host *S. aureus* CCARM 3821 and CCARM 3956 with 10 mM CaCl_2_ supplementation. After overnight incubation at 37 °C, formed single plaques were randomly selected, picked up, and resuspended in SM buffer. Phages recovered from a single plaque (10^7^ PFU/mL) were again challenged 30 times with sodium pyrophosphate. Throughout the challenges, the experimental group’s survival rate was maintained at 1–10% of the control group’s survival rate by incrementally increasing the concentration of sodium pyrophosphate during each challenge up to a maximum of 400 mM. During the final round of challenges, the survival rate of the experimental group nearly reached 100%. Then a single plaque was picked up, propagated, and concentrated as described above to obtain the mutant phage stock.

### Transmission electron microscopy

Prepared phage stocks were spotted on carbon-coated copper grids and negatively stained with 2% aqueous uranyl acetate. Phage particles were observed using a LIBRA 120 transmission electron microscope (Carl Zeiss, Germany) operated at 120 kV. ImageJ was used to measure phage head and tail dimensions (*n* = 5).

### Determination of bactericidal activity of phages

The host *S. aureus* culture (OD_600_ = 0.3) in TSB supplemented with 10 mM CaCl_2_ was infected either with parent or mutant phages at MOIs 1 and 10. The growth of *S. aureus* at 37 °C with aeration was periodically monitored for 17 h by measuring absorbance at 600 nm (A_600_) using a microplate reader (Spark, Tecan, Switzerland). The control group was treated with SM buffer instead of phages.

### Adsorption assay and one-step growth analysis

The adsorption of the phages on bacterial cells was examined as previously described^45^ with some modifications. *S. aureus* cells at the early exponential phase were treated with chloramphenicol (25 μg/ml, final concentration) to cease propagation. Then the phages at an MOI of 0.01 was added to the culture and supplemented with 10 mM CaCl_2_ (final concentration). Adsorption was permitted to occur for 30 min at 37 °C, during which time portions of culture were collected every 5 min and the number of unbound phages were titered by standard plaqueing assay. The titer of phages incubated with TSB instead of the culture was considered as a control, and designated as initial phage titer. The adsorption constant, *k*, was calculated as follows:

$$k = - {\text{ln}}\left( {{\text{P}}_{t} {\text{/P}}_{0} } \right){/}Nt$$

where P_*t*_ is phage titer at time *t* in PFU/mL, P_0_ is the initial phage titer in PFU/mL, *N* is the number of host cells in CFU/mL, and *t* is adsorption time in min.

The phages’ propagation characteristics within individual host cells were examined by one-step growth analysis as described elsewhere^45^ with some modifications. Host bacteria in the early exponential phase were infected with phages at an MOI of 0.01 for 5 min. Then phage-infected cells were harvested and resuspended in fresh TSB. The suspension was incubated at 37 °C with aeration. Two samples were collected every 10 min, one of which was treated with 2% chloroform (v/v, final concentration) to artificially liberate progenies from the bacterial cells. Then samples were diluted with SM buffer. The number of phages was titered through plaqueing assays. The PFU at each time point was divided by the PFU for the samples that were not treated with chloroform at 0 min to calculate the PFU per infected cell.

### Determination of thermal and pH stability

To assess the thermal tolerance of phages, phages in SM buffer (10^6^ PFU/mL) were incubated at 4, 25, 37, 45, 50, 60, 70, and 80 °C for 1 h. To assess their pH stability, phages were mixed with pH-adjusted TSB (pH 2, 3, 4, 5, 6, 7, 8, 9, 10, 11 and 12) and incubated at 37 °C for 1 h. After each treatment, the number of surviving infectious phages was counted by plaqueing assay with the appropriate host bacteria.

### DNA extraction and genomic analysis

Phage genomes were manually extracted from the phage stock using phenol, chloroform, and isoamyl alcohol according to the previously described method^46^ after treatment of DNase (1 mg/mL) and RNase (0.001 mg/mL) to remove external genomic contaminants. Extracted phage genomes were sequenced using Illumina Miseq (Illumina, USA) and reads were de novo assembled with SPAdes v.3.14.1.

Open reading frames (ORF) from the assembled contig was predicted using GenemarkS^47^, FgenesB (Softberry, Inc., Mount Kisco, NY), and Glimmer3^48^. The presence of tRNAs was determined using tRNAscan-SE^49^. The Basic Local Alignment Search Tool (BLAST)^50^ and InterProScan^51^ were used to annotate the gene product of the predicted ORFs with putative functions. TMHMM^52^ and TOPO2^53^ were used to predict and visualize the hydrophobicity and transmembrane topology of TMP.

### Statistical analysis

Relevant data was collected from three independent trials. Statistical analysis was conducted using GraphPad Prism version 8.4.3 (GraphPad Software Inc., USA) and is expressed as the mean and standard deviation, unless mentioned otherwise. A *t*-test was conducted to analyze the independent variables and a *P* value < 0.05 was considered statistically significant.

## Supplementary Information

Supplementary Information.
