# Supplementary material for: Improved bactericidal efficacy and thermostability of Staphylococcus aureus-specific bacteriophage SA3821 by repeated sodium pyrophosphate challenges
Source: Sci Rep. 2021 Nov 25;11:22951. doi: 10.1038/s41598-021-02446-1 (PMC8616913; doi:10.1038/s41598-021-02446-1)
Supplement: Supplementary file 1 — Supplementary Information. [file 41598_2021_2446_MOESM1_ESM.pdf]

Supplementary Information

Improved Bactericidal Efficacy and Thermostability of *Staphylococcus aureus*-specific Bacteriophage SA3821 by Repeated Sodium Pyrophosphate Challenges

Hyoju Choi[1] and Minsik Kim[1, *]

[1] Loboratory of Molecular Food Microbiology, Department of Food and Nutrition, Brain Korea 21 FOUR, Institute of Symbiotic Life-TECH, College of Human Ecology, Yonsei University, Seoul 03722, Korea, Republic of

* Corresponding author.

Mailing address: Department of Food and Nutrition, Yonsei University, Seoul 03722, Korea

Phone: +82 2-2123-3119, fax: +82 2-2123-8661, email: m.kim@yonsei.ac.kr

Table S1. Phage susceptibility of bacterial strains used in the study

Bacterial strains	Genotype	Description	Phage susceptibility[a]			Source
			SA3821	SA3821 [M]	SA3956	
Staphylococcus aureus						
CCARM 3821			++	++	++	CCARM[b]
CCARM 3956			+	+	++	
CCARM 3793		MRSA	-	-	I	
CCARM 3089		MRSA	+	+	++	
CCARM 3090		MRSA	+	+	+	
CCARM 3502			+	+	+	
CCARM 3533			-	-	I	
CCARM 3795			-	-	I	
CCARM 3847			I	I	I	
CCARM 3877			-	-	I	
CCARM 3943			+	+	+	
CCARM 3944			-	-	I	
CCARM 3953			+	+	+	
CCARM 3970			-	-	I	
CCARM 3996			+	+	+	
CCARM 3A008			+	+	+	
CCARM 3A066			+	+	I	
CCARM 3A069			-	-	++	
CCARM 3A165			-	-	+	
CCARM 3A584			-	-	I	
CCARM 3A692			+	+	+	
CCARM 3A817			-	-	I	
CCARM 3A822			+	+	I	
ATCC 33586			-	-	I	ATCC[c]
ATCC 33593			-	-	-	
ATCC 6538			+	+	I	
ATCC 29213			+	+	I	
ATCC 13301			-	-	I	
ATCC 23235			-	-	I	
ATCC12600			-	-	+	
Newman			-	-	+	
RN4220	wild-type		++	++	++	
	Δ*tagO*::*erm*	Loss of wall teichoic acid (WTA)	-	-	-	[1]
	Δ*tagO::erm* complemented	WTA complemented	++	++	++	[1]
	Δ*ltaS::phleo*	Loss of lipoteichoic acid (LTA)	+	+	+	[2]
	Δ*ltaS::phleo* complemented	LTA compemented from pM101-*ltaS*	+	+	+	[2]
	Δ*dltA::erm*	Loss of D-Ala modification of WTA and LTA	+	+	+	[3]
	Δ*ypfP::erm*	Loss of glycolipid	+	+	+	[3]
	Δ*mprF::cm*	Loss of lysyl-phosphatidylglycerol	+	+	+	[4]
	Δ*lgt::erm*	Loss of lipid modification of lipoproteins	+	+	+	[5]
	Δ*spa::phleo*	Loss of Protein A	+	+	+	[2]

	Δ*srtA::cm*	Loss of sortase A	+	+	+	[6]
	Δ*oatA::erm*	Loss of *O*-acetylation of peptidoglycan	+	+	+	[7]

[a], ++, EOP ≥ 1; +, 0 < EOP < 1; I, inhibition zone; -, plaques not formed.

[b]; CCARM, Culture Collection of Antimicrobial Resistant Microbe in South Korea

[c], ATCC, American Type Culture Collection

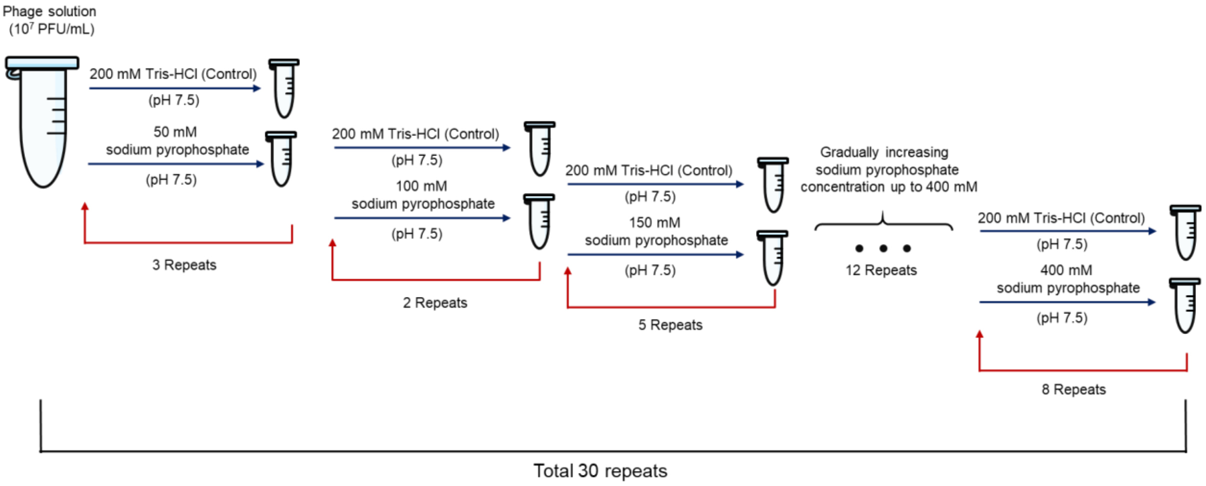

Figure S1. Schematic representation of sodium pyrophosphate challenges. Bacteriophages (10^7 PFU/mL) were treated with the sodium pyrophosphate (50 mM, final concentration; pH 7.5), and the survived phages were treated again with the sodium pyrophosphate at the same or increased concentrations dependent on the relative survival rate of the phages compared to the control (treated with the 200 mM of Tris-HCl). See "*Methods*" for the details.

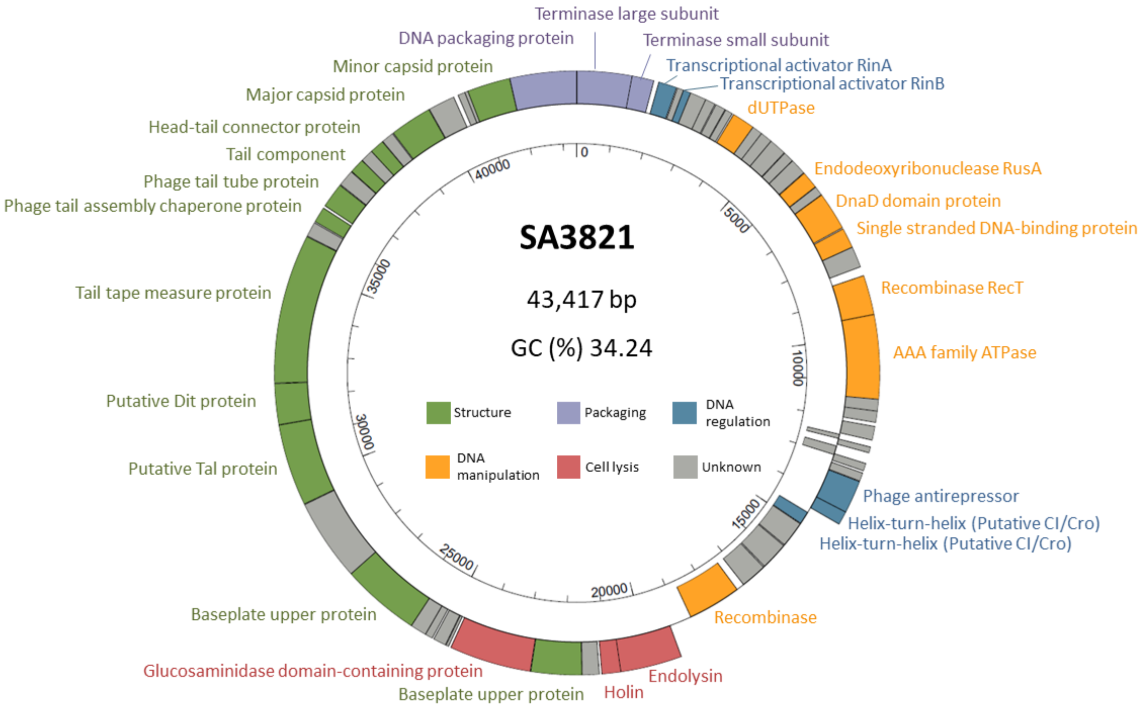

Figure S2. Genomic map of phage SA3821. Complete genome of SA3821 was visualized with a circular map using GeneScene. The color of genes indicates the functional groups: green, structure; purple, packaging; blue, DNA regulation; yellow, DNA manipulation; red, cell lysis; gray, unknown function.

Table S2. Functional characteristics and categorization of predicted ORFs in phage SA3821

Locus (ORF)	Location (nt)	Length (bp)	Predicted function	Functional group
SA3821_01	1..1278	1278	Phage terminase large subunit	Packaging
SA3821_02	1265..1774	510	Phage terminase small subunit	Packaging
SA3821_03	1892..2314	423	Transcription activator RinA	DNA regulation
SA3821_04	2338..2484	147	hypothetical protein	Unknown
SA3821_05	2485..2658	174	Transcriptional activator RinB	DNA regulation
SA3821_06	2658..3044	387	hypothetical protein	Unknown
SA3821_07	3034..3270	237	hypothetical protein	Unknown
SA3821_08	3295..3531	237	hypothetical protein	Unknown
SA3821_09	3548..3721	174	hypothetical protein	Unknown
SA3821_10	3758..4294	537	dUTPase	DNA manipulation
SA3821_11	4287..4544	258	hypothetical protein	Unknown
SA3821_12	4528..4812	285	hypothetical protein	Unknown
SA3821_13	4809..5210	402	hypothetical protein	Unknown
SA3821_14	5223..5471	249	hypothetical protein	Unknown
SA3821_15	5472..5849	378	hypothetical protein	Unknown
SA3821_16	5862..6266	405	Endodeoxyribonuclease RusA	DNA manipulation
SA3821_17	6275..6493	219	hypothetical protein	Unknown
SA3821_18	6500..7387	888	DnaD domain-containing protein	DNA manipulation
SA3821_19	7417..7887	471	Single-stranded DNA-binding protein	DNA manipulation
SA3821_20	7888..8373	486	hypothetical protein	Unknown
SA3821_21	8586..9506	921	Recombinase RecT	DNA manipulation
SA3821_22	9508..11463	1956	ATPase	DNA manipulation
SA3821_23	11460..11723	264	hypothetical protein	Unknown
SA3821_24	11732..11992	261	hypothetical protein	Unknown
SA3821_25	12086..12406	321	hypothetical protein	Unknown
SA3821_26	12407..12574	168	hypothetical protein	Unknown
SA3821_27	12567..12704	138	hypothetical protein	Unknown
SA3821_28	12754..12984	231	hypothetical protein	Unknown
SA3821_29	12959..13207	249	hypothetical protein	Unknown
SA3821_30	13188..13382	195	hypothetical protein	Unknown
SA3821_31	13399..14163	765	Phage antirepressor	DNA regulation
SA3821_32	14163..14477	315	Putative repressor	DNA regulation
SA3821_33	14620..14952	333	Putative repressor	DNA regulation
SA3821_34	14969..15643	675	hypothetical protein	Unknown
SA3821_35	15671..16396	726	hypothetical protein	Unknown
SA3821_36	16428..17108	681	hypothetical protein	Unknown
SA3821_37	17315..18700	1386	Recombinase	DNA manipulation

SA3821_38	19278..20723	1446	Endolysin	Cell lysis
SA3821_39	20704..21141	438	Holin	Cell lysis
SA3821_40	21198..21593	396	Baseplate upper protein	Structure
SA3821_41	21599..22771	1173	Glucosaminidase -containing protein	Cell lysis
SA3821_42	22784..24682	1899	hypothetical protein	Unknown
SA3821_43	24742..24804	63	hypothetical protein	Unknown
SA3821_44	24819..25118	300	hypothetical protein	Unknown
SA3821_45	25159..25335	177	hypothetical protein	Unknown
SA3821_46	25336..25713	378	hypothetical protein	Unknown
SA3821_47	25713..27536	1824	Baseplate upper protein	Structure
SA3821_48	27536..29446	1911	hypothetical protein	Unknown
SA3821_49	29461..31362	1902	Putative Tal protein	Structure
SA3821_50	31371..32318	948	Putative Dit protein	Structure
SA3821_51	32331..35798	3468	Tape measure protein	Structure
SA3821_52	35815..36159	345	hypothetical protein	Unknown
SA3821_53	36189..36554	366	Tail assembly chaperone	Structure
SA3821_54	36616..37197	582	Major tail protein	Structure
SA3821_55	37216..37599	384	hypothetical protein	Unknown
SA3821_56	37611..37958	348	Putative tail-component	Structure
SA3821_57	37958..38260	303	hypothetical protein	Unknown
SA3821_58	38257..38589	333	Head-tail connector protein	Structure
SA3821_59	38598..38885	288	hypothetical protein	Unknown
SA3821_60	38907..39881	975	Phage capsid protein	Structure
SA3821_61	39895..40515	621	hypothetical protein	Unknown
SA3821_62	40624..40794	171	hypothetical protein	Unknown
SA3821_63	40791..40874	84	hypothetical protein	Unknown
SA3821_64	40867..41862	996	Minor capsid protein	Structure
SA3821_65	41869..43407	1539	Phage portal protein	Packaging

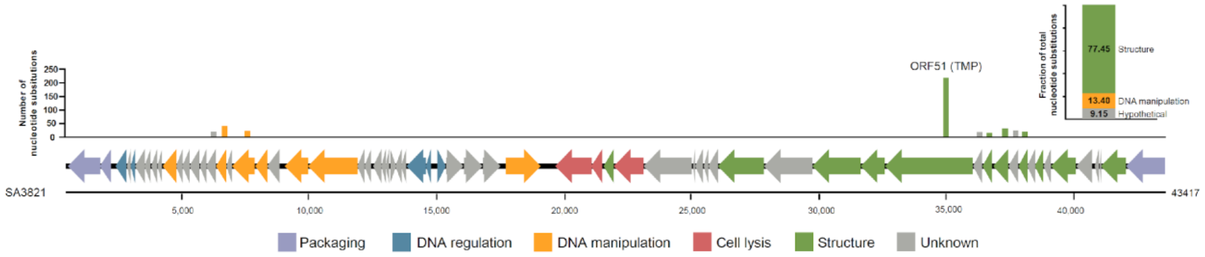

Figure S3. Location and number of mutations observed in SA3821^M. The predicted ORFs of SA3821 were represented as horizontal arrows with the color for each function. The number of mutations at each ORF was represented as bar with the colors correspond to the functions. The fraction of total mutations was analyzed based on the function of the gene where they occurred, and presented at upper-right.

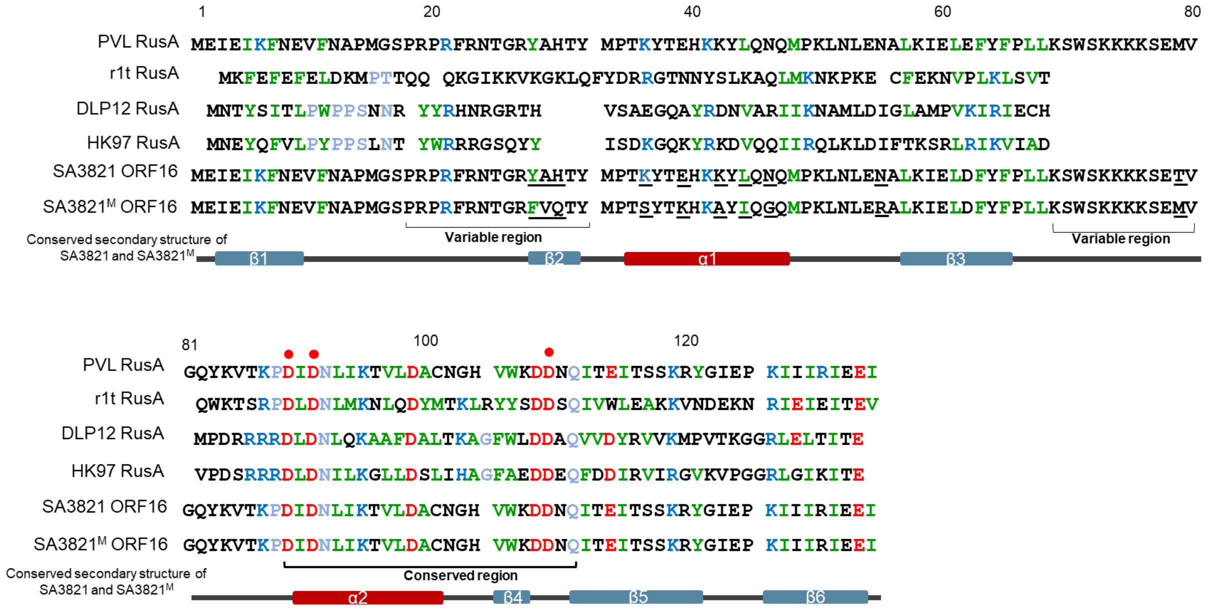

Figure S4. Conserved active site region of RusA (ORF 16). Residues from 1 to 134 of RusA were aligned using ClustalW[8] against homologous sequence from *Staphylococcus aureus* phage PVL (BAA31922.1), *Lactococcus lactis* phage r1t (AAB18689.1), *Escherichia coli* phage DLP12 (CAA63321.1), and HK97 (AAF31142.1). The color of residues indicate highly conserved acidic (red; D), basic (blue; H/K/R), hydrophobic (green; A/F/I/L/M/V/W/Y), and other conserved (skyblue; including Q/N and S/T) residues. Three aspartic acid residues required for catalysis[9] are marked with a red circle. The underlined residues indicate substituted amino acids in mutant phage SA3821^{M}. Flexible regions, and highly conserved region for catalytic function were indicated[10].

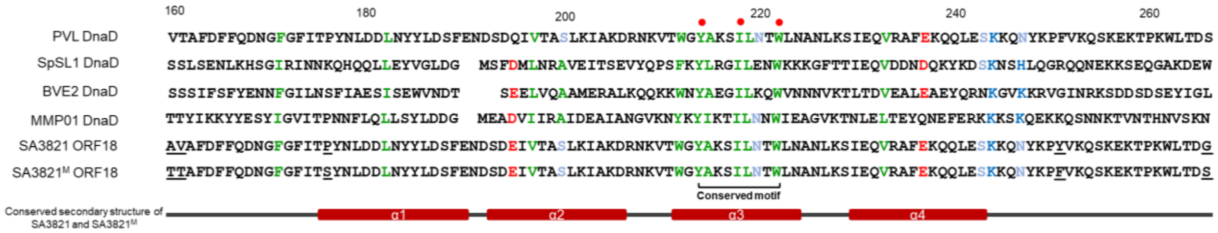

Figure S5. Highly conserved motif at the C-terminal region of DnaD domain (ORF 18). The motif YXXXIXXXW forms secondary helix structure and is reported to have an essential role in DNA binding[11]. Residues from 160 to 266 of DnaD domain containing protein were aligned using ClustalW[8] against homologous sequence from *S. aureus* phage PVL (BAA31920.1), *Streptococcus pneumoniae* phage SpSL1 (YP_009153008.1), *Bacillus cereus* phage BVE2 (AUG88590.1), and *Clostridium difficile* phage MMP01 (YP_009206181.1). Three residues (Y, I, W) required for binding activity are marked with a red circle. The color of residues indicate conserved acidic (red; D/E), basic (blue; H/K/R), hydrophobic (green; A/F/I/L/M/V/W/Y), and other conserved (skyblue; including Q/N and S/T) residues. The underlined residues indicate substituted amino acid of SA3821^{M}.

Table S3. The substituted and deleted amino acids in mutated ORFs

ORFs (predicted gene product)	Type of mutation	Amino acid alteration
ORF 15 (Hypothetical protein)	Missense: 4<br>Silent: 1	K3R, T4K, R9T, H11N
ORF 16 (Endodeoxyribonuclease)	Missense: 10<br>Silent: 9	Y28F, A29V, H30Q, K36S, E39K, K42A, L44I, N46G, N55R, T79M
ORF 18 (DnaD domain)	Missense: 5<br>Silent: 5	A160T, V161T, P176S, Y251F, G266S
ORF 51 (Tail tape measure protein)	Missense: 62<br>Silent: 112	A27S, K30R, Y31F, T34V, K37Q, S44N, T60S, S63A, G65S, E67K, K68N, A69V, K76S, E78Q, K81Q, V84I, A87S, S88N, N92D, Q93K, D95N, V99A, T100S, E106Q, I111T, T115A, T117A, A119V, V129I, N130S, V162I, S168G, N193D, R479H, V500A, V606I, P619A, I620P, S625M, N626D, M628L, G630P, G631L, L632A, G634A, K642L, F643V, L645W, L646V, R647K, A648T, L650A, T651L, I652L, K654S, G655R, F657M, M658T, A821T, I840V, I860M, A865V
	Deletion: 7	I633-, I636-, I637-, A638-, L639-, V640-, S641-
ORF 52 (Hypothetical protein)	Missense: 0<br>Silent: 7	-
ORF 53 (Tail assembly chaperone)	Missense: 1<br>Silent: 2	A114T
ORF 54 (Major tail protein)	Missense: 6<br>Silent: 13	A102S, T103A, S186P, T187P, T189A, G191R
ORF 55 (Hypothetical protein)	Missense: 3<br>Silent: 10	D99E, A104T, I120V
ORF 56 (Putative tail component)	Missense: 1<br>Silent: 7	K103R

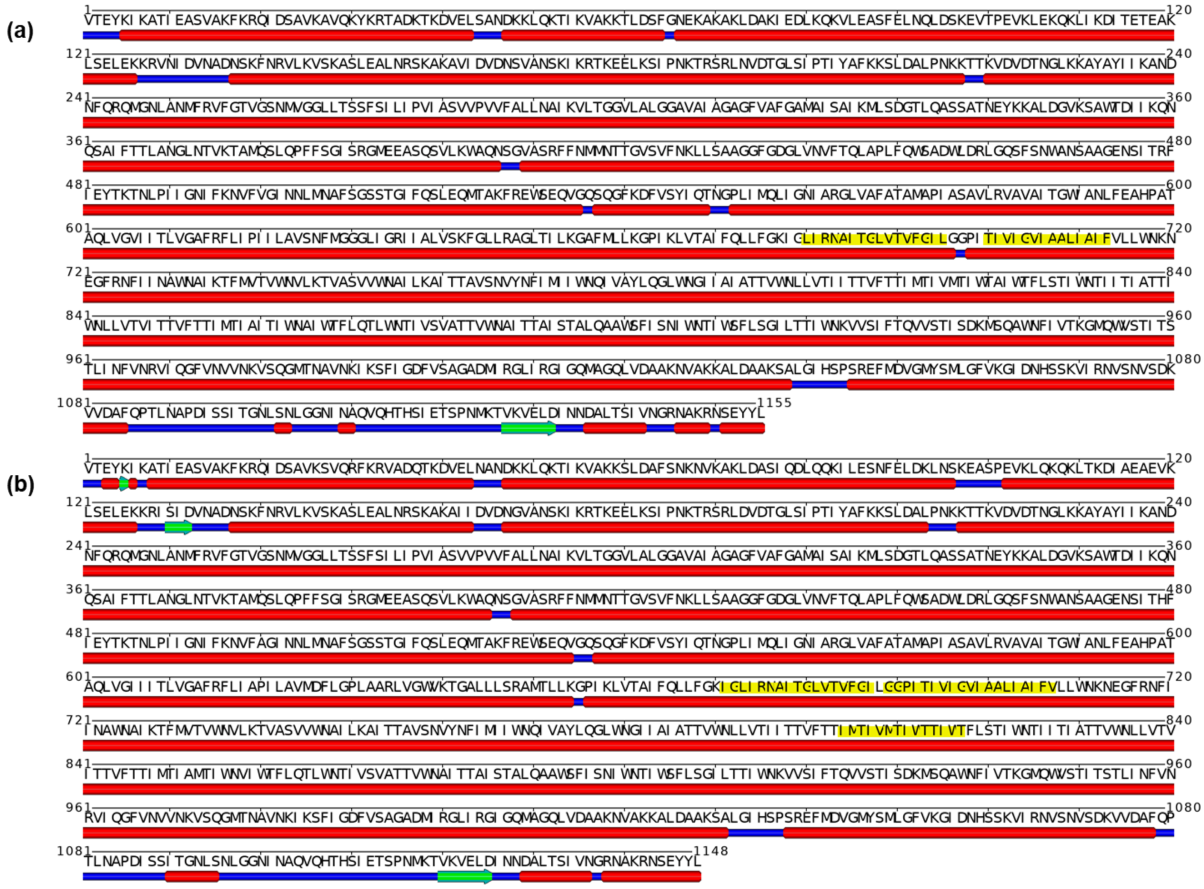

Figure S6. Predicted secondary structure of TMP from **(a)** SA3821 and **(b)** SA3821^{M}. The secondary structure was predicted using SABLE[12] and visualized using POLYVIEW-2D[13]. Each structure was color coded as follows: helices, red; coils, blue; beta-sheets, green; putative transmembrane domain, yellow.

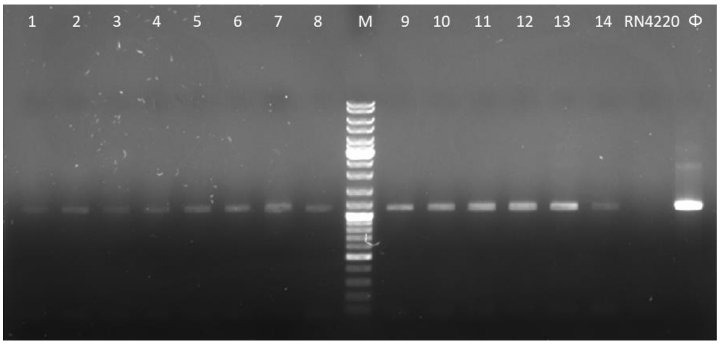

Figure S7. Lysogenization of *S. aureus* with the temperate phage SA3821. Fourteen suspected SA3821 lysogens were isolated and purified from the phage SA3821-treated (MOI=10) *S. aureus* RN4220 culture. Then, they were subjected to the SA3821-specific PCR with primer pairs SA3821_Term_F (5′-GTA CAT TAC GGT GGA GGT TCG -3′) and SA3821_Term_R (5′- ACC GGT CTG TAG AAT CGT TCC-3′) amplifying SA3821’s terminase large subunit. M, 1 Kb plus DNA ladder (Invitrogen); RN4220, *S. aureus* RN4220 wildtype cell as negative control; Φ, phage SA3821 genomic DNA as positive control.

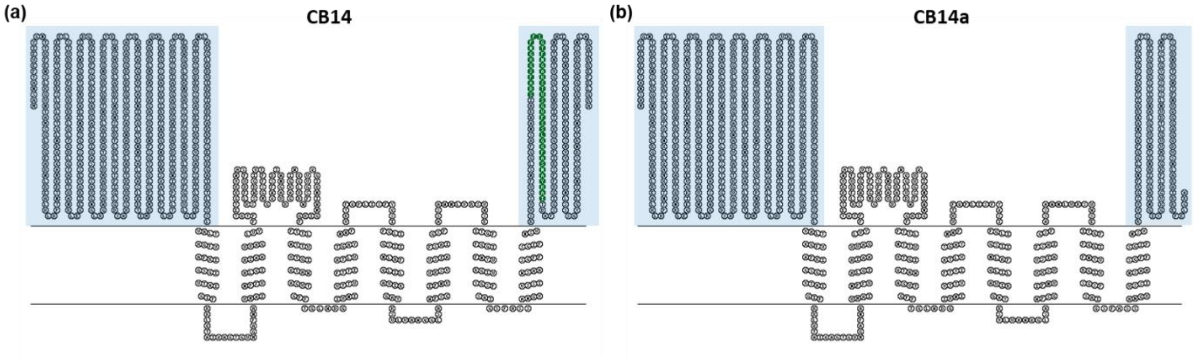

Figure S8. Predicted TMP topology of lactococcal phage CB14 (YP_003127149.1) **(a)** and its thermostable derivative CB14a (ATW69987.1) **(b)**. The 40 a.a. deletion at the TMP C-terminus occurred in the heat-stable mutant phage CB14a was color indicated with green (from S848 to I887) in panel **(a)**. Prediction and visualization of transmembrane region was conducted with TMHMM[14] and TOPO2[15], respectively.

References

1 Xia, G. Q. *et al.* Wall teichoic acid-dependent adsorption of Staphylococcal Siphovirus and Myovirus. *J Bacteriol* **193**, 4006-4009 (2011).

2 Oku, Y. *et al.* Pleiotropic roles of polyglycerolphosphate synthase of lipoteichoic acid in growth of *Staphylococcus aureus* cells. *J Bacteriol* **191**, 141-151 (2009).

3 Kaito, C. & Sekimizu, K. Colony spreading in *Staphylococcus aureus*. *J Bacteriol* **189**, 2553-2557 (2007).

4 Ichihashi, N., Kurokawa, K., Matsuo, M., Kaito, C. & Sekimizu, K. Inhibitory effects of basic or neutral phospholipid on acidic phospholipid-mediated dissociation of adenine nucleotide bound to DnaA protein, the initiator of chromosomal DNA replication. *J Biol Chem* **278**, 28778-28786 (2003).

5 Kurokawa, K. *et al.* The triacylated ATP binding cluster transporter substrate-binding lipoprotein of *Staphylococcus aureus* functions as a native ligand for Toll-like receptor 2. *J Biol Chem* **284**, 8406-8411 (2009).

6 Miyazaki, S., Matsumoto, Y., Sekimizu, K. & Kaito, C. Evaluation of *Staphylococcus aureus* virulence factors using a silkworm model. *Fems Microbiol Lett* **326**, 116-124 (2012).

7 Park, K. H. *et al.* Human serum mannose-binding lectin senses wall teichoic acid glycopolymer of *Staphylococcus aureus*, which is restricted in infancy. *J Biol Chem* **285**, 27167-27175 (2010).

8 Thompson, J. D., Higgins, D. G. & Gibson, T. J. Clustal-W - improving the sensitivity of progressive multiple sequence alignment through sequence weighting, position-specific gap penalties and weight matrix choice. *Nucleic Acids Res* **22**, 4673-4680 (1994).

9 Bolt, E. L., Sharples, G. J. & Lloyd, R. G. Analysis of conserved basic residues associated with DNA binding (Arg69) and catalysis (Lys76) by the RusA Holliday junction resolvase. *Journal of Molecular Biology* **304**, 165-176 (2000).

10 Rafferty, J. B. *et al.* The structure of *Escherichia coli* RusA endonuclease reveals a new Holliday junction DNA binding fold. *Structure* **11**, 1557-1567 (2003).

11 Marston, F. Y. *et al.* When simple sequence comparison fails: the cryptic case of the shared domains of the bacterial replication initiation proteins DnaB and DnaD. *Nucleic Acids Res* **38**, 6930-6942 (2010).

12 Adamczak, R., Porollo, A. & Meller, J. Combining prediction of secondary structure and solvent accessibility in proteins. *Proteins* **59**, 467-475 (2005).

13 Porollo, A. A., Adamczak, R. & Meller, J. POLYVIEW: a flexible visualization tool for structural and functional annotations of proteins. *Bioinformatics* **20**, 2460-2462 (2004).

14 Krogh, A., Larsson, B., von Heijne, G. & Sonnhammer, E. L. L. Predicting transmembrane protein topology with a hidden Markov model: application to complete genomes. *Journal of Molecular Biology* **305**, 567-580 (2001).

15 Moller, S., Croning, M. D. R. & Apweiler, R. Evaluation of methods for the prediction of membrane spanning regions. *Bioinformatics* **17**, 646-653 (2001).
